# Extracellular vesicles in the treatment and prevention of osteoarthritis: can horses help us translate this therapy to humans?

**DOI:** 10.20517/evcna.2023.11

**Published:** 2023-04-17

**Authors:** Thomas J O’Brien, Fiona Hollinshead, Laurie R Goodrich

**Affiliations:** 1 Department of Clinical Sciences, Veterinary Teaching Hospital, Colorado State University, Fort Collins, CO 80523, USA.; 2 Department of Clinical Sciences, Colorado State University, Fort Collins, CO 80523, USA.; 3 Orthopaedic Research Center, C. Wayne McIlwraith Translational Medicine Institute, Department of Clinical Sciences, College of Veterinary Medicine, Colorado State University, Fort Collins, CO 80523, USA.

**Keywords:** Extracellular vesicles, osteoarthritis, equine, animal models

## Abstract

Osteoarthritis (OA) is a common joint disease affecting humans and horses, resulting in significant morbidity, financial expense, and loss of athletic use. While the pathogenesis is incompletely understood, inflammation is considered crucial in the development and progression of the disease. Mesenchymal stromal cells (MSCs) have received increasing scientific attention for their anti-inflammatory, immunomodulatory, and pro-regenerative effects. However, there are concerns about their ability to become a commercially available therapeutic. Extracellular vesicles (EVs) are now recognized to play a crucial role in the therapeutic efficacy observed with MSCs and offer a potentially novel cell-free therapeutic that may negate many of the concerns with MSCs. There is evidence that EVs have profound anti-inflammatory, immunomodulatory, and pro-regenerative effects equal to or greater than the MSCs they are derived from in the treatment of OA. Most of these studies are in small animal models, limiting the translation of these results to humans. However, highly translational animal models are crucial for further understanding the efficacy of potential therapeutics and for close comparisons with humans. For this reason, the horse, which experiences the same gravitational impacts on joints similar to people, is a highly relevant large animal species for testing. The equine species has well-designed and validated OA models, and additionally, therapies can be further tested in naturally occurring OA to validate preclinical model testing. Therefore, the horse is a highly suitable model to increase our knowledge of the therapeutic potential of EVs.

## INTRODUCTION

Osteoarthritis (OA) is a debilitating and common disease afflicting both human and equine populations^[1,2]^. OA is associated with a substantial financial burden and a decreased quality of life and results in loss of athletic performance in humans and horses^[2–4]^. A recent review reported that 303 million people were affected globally in 2017^[5]^. In horses, OA is the most common cause of retirement from athletic activities^[4,6]^. Inflammation is crucial in the disease process through the upregulation of catabolic pathways, ultimately resulting in articular cartilage degradation^[7]^. At a physiologic level, there is a loss in the ability of the joint to maintain normal homeostasis and, invariably, a transition to a catabolic environment. Despite recent advances, no single treatment exists that effectively attenuates joint inflammation, restores the joint to normal health, and promotes adequate regeneration of damaged tissues within the joint. Mesenchymal stromal cells (MSCs) are at the forefront of regenerative medicine, and their purported ability to modulate inflammation and facilitate regeneration in OA has gained increasing momentum^[8]^. However, the use of MSCs as a regenerative therapy for OA is not without significant challenges, including; (i) the underlying ethical concerns associated with the cell source, which is particularly true for embryonically derived tissue^[9]^; (ii) the possibility of undesired tissue formation, reflected by an unstable chondrocyte-like phenotype, or in certain circumstances, teratoma or osteogenic tissue formation^[9,10]^; (iii) the technical and financial demands of harvesting, isolation and expansion of cells in an environmentally controlled facility^[10]^; and (iv) the difficulties associated with upscaling and the lack of consistency across studies. Ultimately it is challenging to conclude the optimal cell source and tissue donor as well as the most efficacious delivery method and the optimal dose of cells required for regeneration^[11]^.

Extracellular vesicles (EVs) are lipid-bound carriers of nucleic acids, proteins, and lipids that are secreted by all cells to affect cellular communication^[12]^. There is now consensus that the EVs produced by MSCs are responsible for the anti-inflammatory and regenerative effects^[13]^. EVs represent a novel cell-free biological therapy that may have similar or superior therapeutic effects to MSCs, yet overcome significant challenges associated with using MSCs as a cell-based therapy^[14]^. EVs have been investigated across many disease syndromes^[13]^, including the OA disease complex^[15]^. OA is ultimately associated with inflammation and articular cartilage degeneration. Administration of EVs has been demonstrated to significantly downregulate inflammatory pathways, ameliorate the progression of OA, enhance healing of critically sized osteochondral defects, improve the quality of repair tissue, and decrease pain in preclinical animal models of OA^[16,17]^. These initial studies provide considerable evidence that EVs have an important role in maintaining normal joint homeostasis and represent a cell-free therapy that may reduce inflammation and promote the healing of articular defects, both of which are considered crucial in the treatment of OA. However, the current limitation of these preclinical studies is that they were predominantly performed either *in vitro* or in small rodent models^[18]^. The horse has been used extensively as a translational model for studying the pathogenesis of OA and the efficacy of various therapeutic interventions over the previous 30 years^[19–21]^. The equine osteochondral chip fragment model of OA performed in the middle carpal joint has been most commonly reported and produces a consistent post-traumatic OA that closely mimics the naturally occurring disease in the horse^[19,20,22,23]^. Preclinical trials using large animal models such as the horse will advance our knowledge of the therapeutic potential of EVs in treating OA in humans. Close translation to humans is possible because of the similarity of joint size, cartilage, and subchondral bone thickness, the ability to serially sample synovial fluid, measure outcomes associated with joint pain, and both arthroscopically and grossly assess joint responses to therapeutics. This review article summarizes the current evidence surrounding the use of MSC-derived EVs for the regeneration of inflamed and injured joint tissue and discusses the pathophysiology of OA with an emphasis on the benefits of using the horse as a translational model.

## THE HORSE AND ITS ROLE AS A TRANSLATIONAL MODEL OF OA

The use of the horse as a model of OA is not novel, and in contrast to small animal models, equine models of OA have several key advantages. Their cartilage and subchondral bone anatomy are more similar to humans compared to other animal models, and naturally occurring primary and secondary (post-traumatic) OA is widespread^[19,24]^. Additionally, using equine models allows for diagnostic imaging, repeated synovial fluid collection, arthroscopic intervention, postoperative management, and the acquisition of large tissue samples^[25,26]^. Indeed, rodent and small animal models may be helpful for initial screening studies; however, large animal models generate more clinically relevant outcomes such as lameness scales and flexion scales, and repeated serial synovial fluid sampling can be performed and are commonly required for regulatory approval of therapeutic interventions in both veterinary and human medicine^[27]^.

A clinically translatable model should have similar anatomical characteristics to humans, particularly the articular cartilage. Anatomic similarities are also important in joint biomechanics, which facilitates clinical translation to humans. In comparison to humans, animal models differ in their gait, and the articular cartilage composition (i.e., thickness) also varies widely. In rabbits, the articular cartilage is approximately ten times thinner compared to humans and has a higher chondrocyte density and zonal cartilage layers that may vary widely within the same joint^[28]^. In rodents, considerable differences are observed between different strains; anatomically, their articular cartilage thickness and composition differ substantially from humans^[28]^. Additionally, the skeletal maturity of rats is often difficult to assess as their growth plates remain open throughout their lifetime^[29]^. Of the animal models, the horse possesses the most similar articular cartilage thickness to humans^[24]^. The cartilage thickness at the distal aspect of the medial femoral condyle in humans has been reported to vary between 1.65 to 2.65 mm^[30]^. In a comparative anatomical study evaluating articular cartilage thickness in the medial femoral condyle across species, the horse had the closest approximation to humans regarding cartilage thickness, ranging from 1.5–2.0 mm^[24]^. This thickness allows for the creation of partial or full-thickness defects, which may be produced to a dimension that most closely reflects chondral/osteochondral lesions in humans^[31]^. Critically sized defects can be created in the articular surface of horses, and the horse is one of the few species in which defect dimensions relevant to humans can be produced^[31]^.

Several experimental equine models of OA have been described [Table 1]. Historically, intra-articular injections of chemicals have been used to elicit joint inflammation. However, there are welfare concerns around the level of induced lameness and the accuracy of OA replication^[19]^. The most recently described model of OA induction in the horse is the osteochondral chip fragment model in the metacarpal phalangeal joint, though further studies are required to document efficacy in its ability to study therapeutics^[25,32]^. To date, the most comprehensively reported equine model of OA is the osteochondral chip fragment model in the middle carpal joint^[19,25,26]^.

### The equine osteochondral chip fragment model of OA

This model produces a clinical syndrome reflective of naturally occurring OA in the horse^[26,33,39]^. It has been extensively validated and utilized to evaluate the efficacy of various therapeutics to treat or limit the progression of OA^[26,33,39]^ [Table 2]. A comprehensive review of this model has been outlined previously^[19]^.

Briefly, an osteochondral fragment is created at the distal-dorsal aspect of the radial carpal bone in one randomly assigned middle carpal joint, while the contralateral joint serves as the unfragmented control^[19]^. In a separate group of horses, a fragment is also created in one carpal joint in the same location and the contralateral limb again acts as the unfragmented control. In one group of horses, fragmented joints act as treated OA joints, and in the other group of horses, fragmented joints act as control OA joints. Horses are housed in a stall and, after a brief postoperative rest period, exercised on a high-speed treadmill that allows for a defined exercise speed and distance. Bi-monthly lameness evaluations are performed throughout the study period for the duration of follow-up. This model allows for clinical, clinicopathologic, biochemical as well as histologic/immunohistochemical analysis of articular tissues frequently involved in the OA disease process^[19]^. Histologic examination of articular tissues has also been described according to the recommendations outlined by the Osteoarthritis Research Society International initiative^[40]^. Drug interventions studied in this model are listed below [Table 2].

## THE ETIOPATHOGENESIS OF OA AND THE ROLE OF INFLAMMATION

The pathophysiology of OA is complex and involves the entire joint, which includes the subchondral bone, ligaments, joint capsule, synovial membrane, and peri-articular tissues^[55–58]^. With the perception to view the joint as an organ, OA reflects failed repair of damage incited to any of these tissues^[58]^. Regardless of the underlying etiology, the consensus is that OA is a whole joint disorder involving cartilage erosion, subchondral bone sclerosis and synovitis^[29,56]^. The role of inflammation, though once questioned, is now recognized to play a pivotal role in the development and progression of OA^[59]^.

The question of what initiates inflammation in OA is still unclear and is likely to differ between endotypes of the disease process (i.e., post-traumatic, age-associated). However, regardless of the inciting cause, the importance of the innate immune system in the inflammatory cycle cannot be understated^[60]^. This system may be activated by interactions between pathogen-associated molecular patterns on pattern recognition receptors of synovial membrane immune cells^[60]^. The most important effect of the innate immune system involves the activation and polarization of synovial macrophages to a predominantly M1 phenotype^[61]^. This macrophage phenotype transition leads to the production of proinflammatory cytokines that have potent downstream signaling effects creating an environment that favors articular cartilage degradation^[61]^.

Two cytokines crucial in the pathogenesis of OA are interleukin-1β (IL-1β) and tumor necrosis factor-alpha (TNF-α) and are produced mainly by synoviocytes, mononuclear cells, and chondrocytes^[62]^. These cytokines induce the expression of matrix metalloproteinases (MMPs), aggrecanases of the ADAMTS (a disintegrin and metalloproteinase with thrombospondin motifs) family, prostaglandin E2 and a multitude of other proinflammatory mediators^[55]^. Of these catabolic molecules, MMP13 and ADAMTS-5 are considered the major enzymes involved in extracellular matrix depletion and cartilage degradation^[55]^. IL-1β and TNF-α also cause downregulation in the production of various anabolic cytokines such as insulin-like growth factor-1 (IGF-1), transforming growth factor-β1(TGF-β1), fibroblast growth factors (FGFs) and bone morphogenetic proteins (BMPs)^[63]^. In physiologic conditions, a balance between anabolic and catabolic pathways exists. In OA, this balance is lost. Therefore, it is crucial that an effective therapy reduces inflammation and restores the joint to a more functional environment that may subsequently facilitate healing.

### Treatment of OA in humans and horses

Articular cartilage is a uniquely hypocellular, avascular, and aneural load-bearing tissue supported by the underlying vascularized subchondral bone^[64]^. The capacity for adequate intrinsic repair is severely limited by its avascular, aneural, and acellular nature. Moreover, regardless of current repair techniques, the neotissue is primarily fibrocartilage, which is functionally inferior to hyaline cartilage^[8]^. Even the smallest chondral/osteochondral defects may significantly alter the normal joint environment, ultimately leading to the initiation and progression of OA. The limited ability of cartilage to heal following injury has led to the development of various therapeutic and surgical interventions in both humans and horses.

An ideal treatment for OA decreases pain, inhibits disease progression, and promotes the regeneration of affected tissues. A gold standard therapy has yet to be identified, and treatment is challenging. Most equine and human patients with OA are managed for disease-associated pain, while the underlying cause is rarely addressed. OA treatments can be broadly categorized into nonpharmacological, pharmacological, and surgical approaches that may have symptom and/or disease-modifying effects^[65–67]^. Nonpharmacological methods commonly include weight reduction, exercise, and physical therapy that focus on restoring or improving the function of early-stage OA patients^[65]^. Pharmacologic therapies in humans and horses frequently include the use of nonsteroidal antiinflammatories drugs (NSAIDs), corticosteroids, glucosamine, chondroitin sulfate, polysulfated glycosaminoglycans and hyaluronic acid (HA)^[68–70]^. Corticosteroids have been the most commonly used pharmaceutical to treat OA in horses for their potent anti-inflammatory and analgesic properties^[68]^. However, treatment with NSAIDs and corticosteroids is not entirely benign, as their use has been associated with gastrointestinal compromise and deleterious effects on cartilage, respectively^[71–73]^. More recently described treatments in horses and humans include the use of anti-nerve growth factor (anti-NGF) monoclonal antibody^[54,74]^, as well as gene therapy to specifically antagonize the effects of IL-1β^[26,75]^. The administration of anti-NGF monoclonal antibody in an equine experimental model of OA significantly reduced synovial membrane inflammation and synovial fluid PGE2 and significantly improved pain^[54]^. Similarly, in humans, the use of anti-NGF in naturally occurring OA provided superior pain relief and improved physical function in patients with knee OA^[74]^. However, the use of a commercially available anti-NGF product is not currently available, and significant expenses are associated with its use.

The use of gene therapy to specifically antagonize the effects of IL-1β has been investigated in both humans and horses to directly attenuate the inflammatory cascade^[26,75]^. Administration of IL-1 receptor antagonist protein (IL-1Ra) was associated with significant improvement in pain, disease activity, and preservation of articular cartilage in an experimental model of OA in horses^[26,76]^. There were consistent reductions in osteophyte formation and the degree of synovitis following treatment, as well as improvements in subchondral bone erosions^[26,76]^. In humans, administration of IL-1Ra was associated with improvements in Knee Injury and Osteoarthritis Outcome Score (KOOS) symptoms and sports parameters in patients with naturally occurring OA^[75]^. Clinical effects following IL-1Ra administration are most likely mediated via reductions in inflammation, which is supported by significant reductions in histological synovitis scores and articular prostaglandin-E2 levels^[26,76]^. However, these treatments are designed to decrease inflammation and improve pain rather than directly promote the regeneration of damaged tissues. In contrast, MSCs and MSC-EVs have been investigated for their ability to facilitate the regeneration of damaged tissues in models of OA^[11,18].^

### The Role of MSCs in treating OA in the human and equine patient

The use of MSCs has gained significant interest in the regeneration of damaged tissues across various disease syndromes. Although regenerative medicine has progressed substantially, studies continue to differ in their designation of the acronym MSC. In a position statement by the International Society for Cellular Therapy, the term mesenchymal stem cell should be reserved for a subset of plastic-adherent cells that express specific surface molecules and are capable of differentiating into specific, multiple cell types *in vitro*^[77,78]^. They propose to refer to the plastic-adherent cells commonly referred to as stem cells as multipotent mesenchymal stromal cells; the same acronym applies; however, the definition of MSC is clarified.

Several review articles have discussed the emerging role that MSCs may play in cartilage regeneration and OA in humans, as well as the mechanisms involved underlying their therapeutic properties^[8,67,79–81]^. Briefly, these studies may be separated into *in vitro*, animal studies (preclinical), and human studies (clinical)^[8]^. *In vitro* studies offer the advantage of a qualitative and quantitative assessment of explants through histology, immunohistochemistry, qPCR, biochemical analysis, imaging, and mechanical testing^[8]^. However, these studies may lack clinical translation. This is true for some preclinical studies, while clinical studies lack the ability to evaluate large samples of the neotissue architecture and rely primarily on improvements in clinical scoring parameters^[81]^.

The majority of OA *in vitro* studies involve bone marrow (BM) derived MSCs stimulated with TGF-β1, delivered in a natural scaffold to evaluate the regenerative capacity of cartilage following injury^[8]^. Most preclinical studies have investigated the efficacy of BM-MSCs in downregulating inflammation and proinflammatory pathways, increasing anabolic signaling, improving the quality of repair tissue in defect models, decreasing pain, and ultimately, limiting the progression of OA^[8,11]^. Clinical studies commonly focus on the capacity of BM-MSCs to improve predefined scoring parameters, including radiological assessment (i.e., osteophytosis), subjective evaluation of tissue healing via arthroscopy, or decreased pain in patients with knee OA^[8]^. However, it must be noted that there is extreme heterogeneity between study designs. *In vitro* and preclinical studies differ considerably in the species, cell source, type of scaffold, and method of MSC stimulation, and assessment of outcome parameters are also not similar.

Similarly, in clinical studies, there is an inconsistency between the tissue origin, dose of MSCs, method of delivery, cell expansion stage, and rehabilitation protocols^[8]^. In a recent review evaluating the potential of MSCs for treating knee OA and chondral defects in humans, there was an overall improvement across studies in self-reported physical function and significant improvements in cartilage volume^[82]^. In contrast to humans, there are limited reports detailing the use of MSCs for the treatment of OA or joint injury in horses^[48,83,84]^. Equine studies differ in their source of cells, the location of OA, and the type of joint injury^[48,83–85]^. In horses with naturally occurring OA of the metacarpal phalangeal joint, administration of chondrogenic-induced MSCs was associated with a significant improvement in lameness compared with controls^[84]^. In a study on femorotibial joint disease, a greater proportion of horses with meniscal injuries returned to work after treatment with BM-MSCs compared with previous reports^[83,84]^. Initial results in horses and humans for using MSCs in OA are promising. However, the heterogeneity across study designs leads to a degree of uncertainty in their use. While most studies report a low incidence of complications associated with administration, it is crucial to consider the potential challenges related to MSC therapy for OA.

## MECHANISM OF ACTION OF MSCS AND THE ROLE OF EVS

The efficacy of MSCs was initially proposed to be attributable to their ability to differentiate into various types of specialized tissues (i.e., chondrocytes)^[79]^. However, this theory has been redefined, and there is increasing evidence that MSCs also have an important role in immunomodulation and paracrine signaling^[79]^. It is postulated that the functional benefits observed after MSC administration in experimental models of tissue injury are more likely related to the production and secretion of bioactive factors^[79]^. Ultimately, these are believed to modulate the injured tissue environment and organize subsequent regenerative processes facilitated through cell migration, proliferation, and differentiation^[86]^. The release of soluble factors underscores the paracrine signaling effects of MSCs, which mediate inter-cellular communication^[87]^. In MSC culture and expansion, many of these soluble factors are released into the conditioned medium. Several studies have shown that the administration of cell-free conditioned media obtained from MSCs has similar reparative effects to MSCs alone^[14,88,89]^. Historically, pivotal studies in EV development and progression were focused on the cardioprotective effects of MSC-conditioned medium in ischemic models of cardiac injury^[14,89]^. First, administration of MSC-conditioned medium had similar cardioprotective effects to MSCs alone, and second, it was shown that EVs were the main soluble factor present in MSC-conditioned medium responsible for therapeutic efficacy^[14,89]^. It is now widely accepted that EVs are the main soluble factor released by MSCs and are always present in MSC-conditioned medium^[14]^. The fact that MSCs show promise for the treatment of OA and that EVs are responsible for the therapeutic efficacy observed with MSC treatment has led to a growing body of evidence for the use of EVs in OA^[90,91]^.

### Challenges associated with the use of MSCs in the treatment of OA

Increasing scientific attention toward EVs in OA therapies originates mainly from the inherent challenges associated with using MSCs as a therapeutic. There are several challenges regarding the use of MSC therapy for OA. Firstly, there are concerns with the use of cell-based therapy for the treatment of OA, including potential immunological rejection, risk of teratoma formation, and ethical considerations regarding acquisition^[9,92]^. Generally, MSCs are considered to have minimal immunogenicity due to the low expression of MHC-1 and HLA-1 and the lack of expression of other costimulatory factors^[93]^. However, if the amplification process and culture conditions are not maintained appropriately, MSC immunogenicity may increase^[10,93]^. Secondary immunogenicity may be related to *in vivo* positive feedback loops and could explain the lack of efficacy reported in some clinical trials. One of the biggest challenges that MSC therapy poses for OA is the difficulty of differentiated MSCs to maintain a stable phenotype. In some instances, the phenotype may shift from chondrogenic to hypertrophic, which generally precedes osteogenesis^[94]^. Additionally, MSCs have a limited replicative lifespan once they transition into a state of senescence, and their beneficial effects may therefore be short-lived^[10]^. Ethical considerations for the use of MSCs must be addressed, and widespread controversy remains surrounding the use of ESCs^[9]^. The biggest current limitations are the considerable heterogeneity across studies as well as the use of allogeneic sources. Despite extensive research, one of the most critical issues in allogeneic MSC therapies is the choice of the donor, as the donor’s overall health and age may affect MSC potential^[10]^. Any underlying recipient comorbidities further complicate this (for instance, diabetes in people), which may strongly influence signaling pathways^[10]^. Additionally, FDA approval for cell-based therapies involves years of research, and the ability to commercially upscale MSCs is wrought with expense, difficulty, and regulatory concerns. The use of MSCs as a regenerative therapy for humans and horses with OA is still in its infancy. However, there is a shift of focus towards MSC-EVs which present as a cell-free therapy with similar efficacy and great potential to overcome many of the challenges that face the use of MSCs.

## EXTRACELLULAR VESICLES

EVs are critical to intercellular communication over short- and longer-range signaling events^[13]^. They carry a cargo of nucleic acids, proteins, and lipids reflecting their cellular origin and are released into the extracellular space by nearly all cells during both physiologic and pathologic conditioning^[13]^. EVs have been isolated from various bodily tissues, including blood, milk, saliva, malignant ascites, amniotic fluid, and urine^[95]^. EV composition should not be considered a mere duplicate of cytosolic content but rather as carriers of specific proteins and nucleic acids that are selective and packaged to deliver therapeutic cargo to tissues.

**Figure 1.**
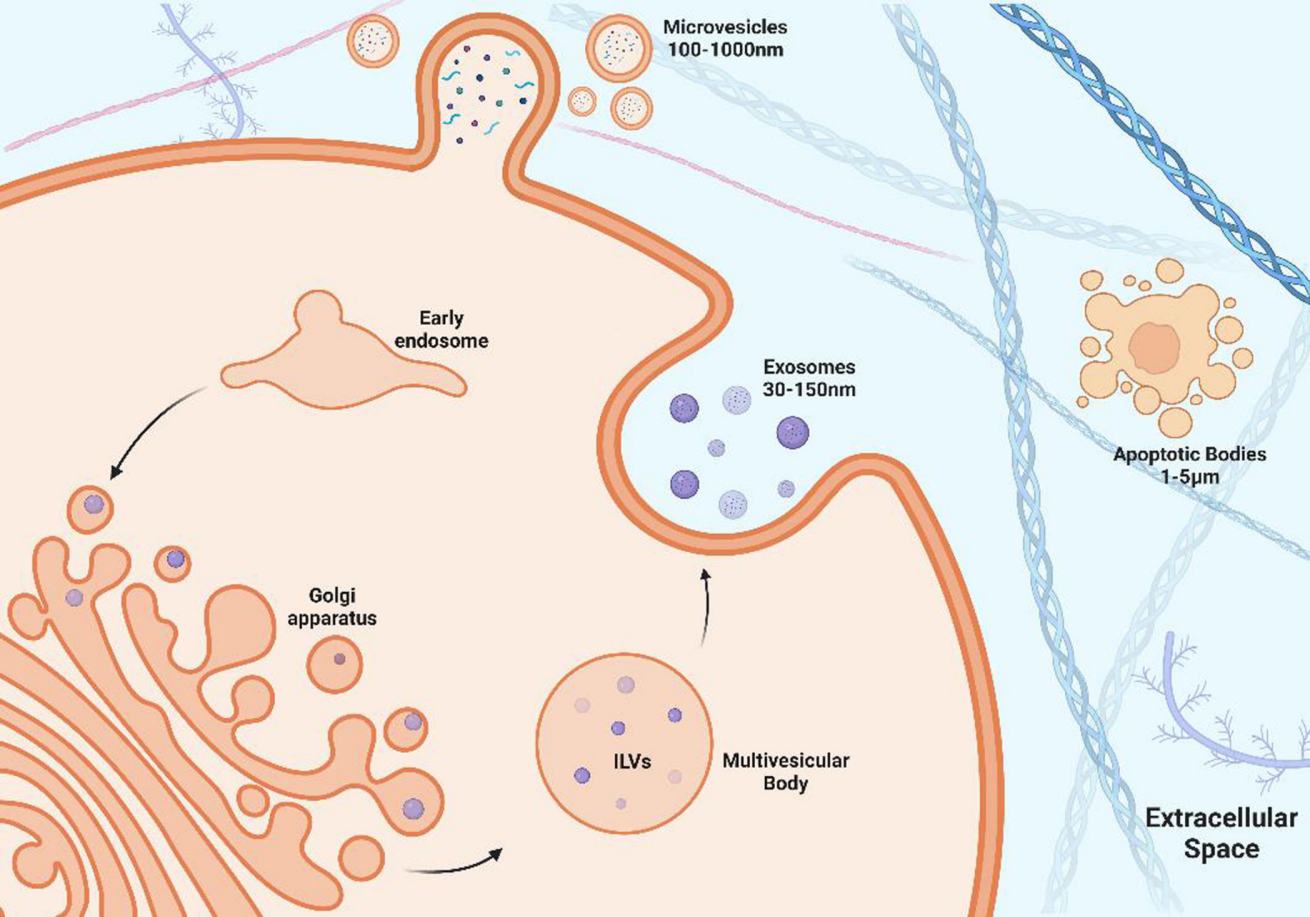
Biogenesis of EVs. Exosome formation begins with the invagination of the plasma membrane to form an early endosome. The early endosome internalizes proteins and a variety of RNA that are packaged by the golgi apparatus, forming intraluminal vesicles (ILVs) within a multivesicular body (MVB). The MVB travels to the cell membrane to release ILVs, which are then referred to as exosomes. Microvesicles (MVs) are formed by the packaging of proteins and RNA at the level of the cell membrane by the process of outward budding.

EVs can be broadly categorized into three distinct groups based on their biogenesis and size. Discrete biogenesis pathways form exosomes, microvesicles (MVs), or apoptotic bodies [Figure 1]. All three EV types contain a bilayer lipid membrane surrounding a specific cargo of biomolecules (i.e., proteins, RNA, and cellular debris)^[13]^. It is their size and respective mode of biogenesis that distinguish them from one another. Exosomes are the most widely studied, are approximately 30–150 nm in diameter, and are formed by inward budding of endosomal membranes, resulting in the progressive accumulation of intraluminal vesicles (ILVs) within large multivesicular bodies (MVBs)^[95]^. In contrast, MVs are much larger in diameter, approximately 100–1,000 nm, and are released by outward budding of the plasma membrane. The cell constituents of MVBs are less well-defined than exosomes^[96]^. Apoptotic bodies are approximately 50–5,000 nm in diameter and are classified as heterogeneous vesicles that are released from cells undergoing apoptotic cell clearance^[97]^.

### Molecular composition and cell sources of EVs

The cargo of EVs is incredibly complex, consisting of specific sorted proteins, lipid derivatives, and abundant miRNA surrounded by a lipid bilayer membrane which protects the contents from the harsh extracellular environment^[13]^. In recognition of the complexity and variation in molecular composition, the International Society for Extracellular Vesicles (ISEV) was established to regulate studies and the classification of EVs. In 2018, a position statement of the ISEV was established to define minimal information for studies of extracellular vesicles (MISEV)^[98]^. The collaboration of ISEV, MISEV, and online databases (i.e., EV-TRACK) has increased knowledge and understanding of the luminal cargo of EVs.

The physiologic and pathologic functions of EVs are closely related to their cell of origin. The molecular composition of EVs may be affected by their cell of origin and is an important consideration, particularly if a therapy is to be somewhat standardized and controlled. While numerous cells produce EVs, OA-related research in horses and humans has almost entirely focused on MSC-EVs^[91]^. These studies have included EVs derived from BM, adipose tissue, umbilical cord, synovial membrane/fluid, embryonically derived EVs, and EVs isolated from induced pluripotent stem cells (iPSC-EVs)^[18]^. This brings into question the effect of the MSC’s potency on EV efficacy. Adult MSCs have little capacity to differentiate into tissue types than other stem cell types. Additionally, MSC-EVs sourced from young individuals promote osteogenic differentiation and inhibit osteoclast formation^[99]^. In contrast, adult MSC-EVs may cause adipogenic differentiation and activate osteoclasts^[99]^. However, the acquisition of adult MSCs is easier, and ethical and legal implications involved with other cell types may be overcome. Therefore, most studies have isolated EVs from adult MSCs. There is a lack of data comparing the potency of different MSC-EVs in the treatment of OA. However, in one study, greater therapeutic efficacy was observed for iPSC-EVs compared to synovial membrane-derived EVs in the treatment of OA^[100]^. It was proposed that induced MSC-EVs increased stimulation of chondrocyte migration and proliferation^[100]^. At least in the context of adult MSC-EVs, human adipose-derived MSCs EVs (ADMSC-EVs) may differ from BMMSC-EVs in molecular composition and functional effects^[101]^. In horses, the molecular composition of ADMSC-EVs was shown to vary when acquired from a single source^[102]^, and BMMSC-EVs demonstrated improved anti-inflammatory properties compared with synovial fluid-derived EVs in an *in vitro* model of inflammation^[103]^. The relative heterogeneity between studies further complicates the influence of cell sources on EV efficacy. While most studies evaluate the effects of exosomes in OA or osteochondral regeneration, several studies have used MVs^[18,104,105]^. In one study, BMMSC exosomes and MVs were equally as effective in attenuating inflammation *in vitro* and ameliorating OA *in vivo*^[105]^. However, the same authors also showed that BMMSC exosomes were more efficient than MVs in suppressing inflammation *in vivo* in a similar model of inflammation^[106]^. The observed differences, although not entirely clear, are proposed to be due to variations in immunomodulatory effects between different EV subsets. However, further studies are required for clarification. Additional sources of EVs that have been described include amniotic and embryonically derived EVs^[107–109]^. These sources of EVs are not as well described as BMMSC-EVs or ADMSC-EVs. However, there is increasing evidence showing they play an important role in maternal cross-talk and immunomodulation and also possess potent anti-inflammatory properties that are similar to their parent MSC^[107–109]^. Bacterial EVs are also naturally occurring and have been investigated for treating bone disease (i.e., osteoporosis)^[110,111]^. However, there is concern that naturally occurring EVs may have insufficient targeting ability and poor therapeutic efficacy^[110]^. More recently, there has been an emerging focus on engineered EVs to increase more precise control and specificity of protein and RNA delivery to target cells^[112,113]^. Regardless of the source of MSC-EV or the type of EV, there is considerable evidence that EVs isolated from adult stem cells efficiently reduce inflammation, and facilitate healing in experimental models of OA, equally or more effectively than their parent MSC^[105,106]^.

EV intercellular communication and uptake mechanisms are essential in their ability to deliver their cargo. Broadly, EVs may directly interact with extracellular receptors, fuse with the plasma membrane, or be internalized^[114]^. The EV uptake mechanisms involve protein interactions that subsequently facilitate the route of interaction. The use of fluorescent microscopy has allowed for direct visualization of EV uptake, and it is proposed that internalization of the EV by the recipient cell is the most common uptake method^[115]^. Once internalized, the EVs undergo uncoating and fuse with endosomes, allowing for the release of their cargo^[114]^. EVs may also fuse with the plasma membrane and release their content directly into the cytosol of target cells or interact with cell surface receptors on the recipient cell generating downstream signaling^[114]^.

### Potential advantages to using EVs for OA

EVs are a novel, cell-free therapy with equal or superior efficacy to MSCs in the treatment of OA^[91,105,106]^. As a result, their potential role as a replacement for cell-based therapy to treat OA is rapidly accumulating evidence. Compared to MSCs, one of the main benefits of using EVs is that they are a cell-free therapeutic. This negates the requirement to administer a cell-based therapy and should assist EVs in overcoming many of the regulatory obstacles and ethical concerns that challenge MSCs. Additionally, their small size and their lipid membrane not only provide stability but help to prevent degradation. So far, across studies, no side effects have been reported, which suggests that their administration may be safe, and they have reduced immunogenicity compared with MSCs, although this still needs to be proven through rigorous safety studies^[90]^. Unlike MSCs, EVs do not have additional procedures necessary for culture expansion or delivery^[90]^. With appropriate isolation procedures, current evidence suggests that EVs may be stored for extended periods at −80 °C^[116]^. The ability to remain stable over time makes EVs a potential off-the-shelf therapeutic. With the development of more efficient manufacturing processes, such as tangential flow filtration coupled with chromatography-based methods, upscaling with current-Good Manufacturing Practices for therapeutic purposes may not be an impossible accomplishment in the near future^[117,118]^.

### The role of EVs in inflammatory signaling in OA

EVs facilitate communication between cells of different origins and are proposed to have a crucial role in inflammatory signaling pathways in OA^[119,120]^. During joint inflammation and disease progression, infiltrating leukocytes and resident synovial macrophages are thought to activate fibroblast-like synoviocytes in the synovial membrane through EV-mediated communication^[121]^. Activated synoviocytes maintain joint inflammation and release EVs that confer inflammatory signals to immune cells, thereby modulating the release of cytokines and enzymes^[122]^. EVs isolated from OA-affected chondrocytes, as well as inflamed chondrocytes and synoviocytes, can directly upregulate proinflammatory signaling cascades^[123–125]^.

EVs isolated from chondrocytes of OA-affected joints enhanced the production of mature IL-1β by macrophages^[123]^, and EVs derived from chondrocytes treated with IL-1β resulted in nearly a 3-fold increase in MMP-13 compared to chondrocytes without IL-1β stimulation and upregulated the production of IL-1β, TNF-α, and cyclooxygenase-2 (COX-2) from the synovial membrane^[124]^. Additionally, EVs from synovial fibroblasts stimulated with IL-1β caused a significant increase in MMP-13, ADAMTS-5 expression in chondrocytes, and downregulation of COL2A1 and ACAN^[125]^. These studies highlight that the function of EVs may change based on the overall health of the parent cell and that EVs are closely involved in proinflammatory signaling events that are important in OA.

While EVs may play a role in the upregulation of inflammation in OA-affected joints, there is considerable evidence that they also have potent anti-inflammatory and immunomodulatory effects in similarly inflamed tissues^[16,103,126,127]^. Most studies treat chondrocytes with IL-1β, a model that reliably produces an inflammatory response. In rats, the administration of BM-MSC-derived EVs to IL-1β stimulated chondrocytes significantly attenuated the inhibitory effect of IL-1β on the proliferation and migration of chondrocytes^[126]^. This study also found that EV treatment significantly increased the expression of COL2A1 and ACAN and reduced the expression of MMP13 and ADAMTS5^[126]^. In an inflammatory *in vivo* OA model, EV treatment significantly upregulated COL2A1, downregulated MMP13 production, and significantly improved pain scores in rats^[126]^. In a study using human OA cartilage explants, administration of BMMSC-EVs dampened TNF-α upregulation of COX2 and other proinflammatory cytokines and inhibited TNF-α induced collagenase activity^[128]^. In a more recent study, treatment with human umbilical cord MSCs (hUMSCs) derived EVs effectively promoted the polarization of macrophages towards an M2 phenotype, and treatment with the supernatant from EV stimulated M2 macrophages upregulated anabolic gene expression and downregulated MMP-13 and TNF-α production on IL-1β stimulated chondrocytes^[16]^. In horses, the delivery of equine BM-MSC-derived EVs was shown to significantly reduce inflammatory markers associated with OA in chondrocytes treated with IL-1β^[103,129]^. Inflammation is considered a key component in the pathogenesis of OA, and these initial studies highlight that EVs have potent anti-inflammatory and immunomodulatory effects in inflamed and OA-affected tissues.

### The role of EVs in cartilage and osteochondral regeneration

The potential role of EVs as a therapeutic for the treatment of OA extends well beyond their anti-inflammatory effects. There is a growing body of research evaluating the potential role of EVs in facilitating osteochondral and/or chondral regeneration in experimentally induced defects, or OA-affected chondrocytes *in vitro*^[18,130]^. An overlap generally exists between *in vitro* studies that evaluate inflammation and regeneration of OA-affected tissues, as these usually occur concurrently. In one study, chondrocyte explants taken from naturally occurring OA-affected human knees and treated with allogeneic BMMSC-EVs demonstrated cartilage regeneration and increased production of proteoglycans and type II collagen^[128]^. In another study, the administration of human BMMSC-EVs to IL-1β stimulated chondrocytes, suppressed chondrocyte apoptosis, and protected against degradation^[130]^.

Most preclinical studies have used rats or mice, in which osteochondral defects were surgically created and designated as either a control or treated joint. EVs are most commonly acquired from BM and delivered articularly, while the control treated joint varies between studies^[18]^. Across studies, outcome parameters consistently compare the quality and the ability of repair tissue to integrate with adjacent tissue in the treated joint relative to the control joint^[18]^. In most studies, EV-treated joints show increased cellular proliferation, enhanced matrix deposition, and improved histological scoring compared with controls^[17,18,128,131]^. Osteochondral defects created in the trochlear groove of rats that were treated with EVs demonstrated superior healing compared with controls (phosphate buffered saline), and by 12 weeks, there was: (i) complete restoration of cartilage and subchondral bone with hyaline cartilage; (ii) complete integration with the adjacent articular cartilage; and (iii) an extracellular matrix composition that closely resembled that of age-matched unoperated controls^[17]^. Other preclinical studies in rodents demonstrate consistent findings; that articular delivery of MSC-EVs into a joint with a surgically created osteochondral defect promotes superior healing compared with control-treated joints^[17,128,131]^. In addition to rodent models, EVs also facilitate osteochondral/chondral repair in relevant porcine and rabbit models of osteochondral damage^[132,133]^. In the study by Zhang *et al*., 2022, osteochondral defects were created in the medial femoral condyle in micropigs, and joints were either treated with either MSC-EVs and HA or HA alone^[132]^. The administration of MSC-EVs produced significantly better MRI scores and functional cartilage and subchondral bone repair, with significantly improved macroscopic and histologic scores and biomechanical properties^[132]^. In the study by Yang *et al*., 2022, chondral defects were created in the patella groove of rabbits. Administration of human BMMSC-EVs facilitated cartilage regeneration, improved proteoglycan content, and increased the International Cartilage Repair Society score^[133]^.

The ability of EVs to facilitate osteochondral regeneration is most likely attributable to their immunomodulatory effects. Chondrocytes rapidly internalize EVs and, at least in part, activate receptor-mediated signal transduction via phosphorylation of survival kinases such as AKT and ERK and favor a higher infiltration of M2 macrophages over M1 macrophages^[131]^. To date, the efficacy of EVs in treating OA in the horse is limited only to *in vitro* studies.

### Current challenges to using EVs for joint-based therapies

Despite the fact that there are numerous studies providing evidence for the use of EVs in OA, there remains some degree of heterogeneity between studies in the isolation and characterization of EVs used. Though there is a significant push by the ISEVs body to have more standardized criteria, further studies that have more homogenous isolation, analysis, and delivery methods are required to create some degree of consistency. Additionally, there is little information evaluating the optimal source of cells, stage of differentiation, and method of delivery, all of which are likely to be important in the treatment of OA and the development of a commercial product. The optimal delivery method cannot be understated, as there are challenges associated with acquiring a sufficient number of EVs in *in vivo* and human clinical trials^[122]^. This may be problematic with systemic delivery, where an increased number of EVs may be required for a local therapeutic effect and overcome with regional (i.e., intra-articular) delivery. Further information is required regarding the biodistribution of EVs and, more specifically, the site of action. Biodistribution is complex and may be influenced by the cell source, route of administration, and targeting^[134]^. Currently, there are only several clinical trials evaluating the efficacy of EVs across a range of disease conditions^[135]^. Most of these clinical studies focus on biomarkers, pathological mechanisms, and cancer treatment, and only a small number evaluate the efficacy of EVs in the context of a joint-based therapy. Although there is evidence supporting the use of EVs as a potential treatment for OA, another consideration is that the specific mechanisms by which EVs promote tissue repair and regeneration have yet to be fully elucidated. Although there is increasing evidence that miRNAs are important in mediating therapeutic effects, further studies are required to discern the mode of action^[136]^. This is an important question as it may facilitate a targeted and more specific treatment. Many of these unanswered questions are likely to be answered in the future with more consistent and standardized protocols.

## CONCLUSIONS

The use of EVs in treating joint disease is in its infancy. Initial results across *in vitro*/*in vivo*/preclinical/clinical/ studies demonstrate that EV administration has potent anti-inflammatory and pro-regenerative effects and enhances the qualitative and biomechanical properties of reparative tissue, a process most likely mediated through immunomodulation. Therefore, EVs are an exciting potential therapeutic for the treatment of OA, with their efficacy demonstrated in these early, preliminary OA preclinical studies. The acellular nature of EVs offers clinical safety and long-term storage/immediate use potential. Most preclinical studies evaluating EV use in OA are confined to small animal rodent models. While these preclinical studies are a necessary first step in documenting safety and efficacy, translating these findings to humans has significant limitations. Future studies should focus on using a validated, consistent large animal model. Of those described previously, the horse may offer the most clinically relevant model to allow close translation to humans.

## Supplementary Material

Video

## Figures and Tables

**Table 1.** Preclinical models of OA induction described in the horse

Model of OA induction	Joint involved	Length of follow up	Population size	Outcome/Findings	Year
**Carpal osteochondral fragment model** ^[19,33,26,34]^	Middle carpal	70 days	16	Lameness; effusion; increased radiographic scores of OA; Increased Col I; elevations in PGE-2; cartilage erosions; mild synovitis; reduced proteoglycan content of cartilage	1994 - present
**Transection of collateral and lateral collateral ligaments** ^[35]^	Metacarpophalangeal	8 weeks	6	Lameness; effusion; osteophytes; articular cartilage erosion; wear-lines	1999
**Single impact on the medial femoral condyle** ^[36]^	Medial femorotibial joint	84 and 180 days	10	Early decrease in synovial fluid GAG; macroscopic and microscopic articular lesions	2006
**Non-terminal osteochondral fragmentation** ^[37]^	Metacarpophalangeal	16 weeks	11	Joint effusion; enthesophytes; superficial chondrocyte death	2013
**Osteochondral fragment-groove** ^[32]^	Metacarpophalangeal	77 days	6	Lameness; increased synovial fluid IL-6, PGE-2, HA and IL-1Ra	2019
**Osteochondral fragmentation** ^[25]^	Metacarpophalangeal/metatarsophalangeal	12 weeks	8	Joint effusion; wear-lines; macroscopic and microscopic cartilage damage; increased Col I	2020
**Equine talar impact model** ^[38]^	Tarsocrural joint	6 months	8	Reduced bone sclerosis, cartilage fibrillation; increased PGE-2	2020

GAG: Glycosaminoglycan; Col I: collagen type 1; PGE-2: prostaglandin E2; HA: hyaluronic acid; IL-1Ra: interleukin 1 receptor antagonist protein; IL-6: interleukin 6.

**Table 2.** Carpal osteochondral fragment model of OA induction in the horse and evaluated treatments

Treatment/Intervention	Length of follow up	Population size	Outcome/Findings	Year
IA Betamethasone^[41]^	70 days	12	No difference in histopathological scoring between OA treatment and OA control joints, and no detrimental effects of treatment in OA joints	1994
IA Triamcinolone^[21,42]^	70 days	12	Reduced lameness in treated joints; improved articular histological parameters and synovial membrane parameters in treated joints	1998, 1996
IV Sodium hyaluronate^[20]^	70 days	12	Reduced lameness in joints of treated horses; reduced total protein and PGE-2 in joints of treated horses 72 h after surgery	1997
IA 6α-Methylprednisolone acetate^[43]^	70 days	18	Reduced PGE-2, intimal hyperplasia, and vascularity but increased articular erosion and morphologic lesions in treated joints	1998
IA Interleukin-1 receptor antagonist protein^[26]^	70 days	16	Reduced lameness in treated joints; Reduced effusion; Reduced gross pathologic changes in treated joints; Reduced synovial vascularity; Improved proteoglycan content	2002
IA Autologous conditioned serum^[44]^	70 days	16	Reduced lameness in treated joints; Reduced synovial membrane hyperplasia in treated joints; Reduced gross articular fibrillation in treated joints as well as increased synovial fluid IL-1Ra concentrations	2007
Oral Avocado/Soybean^[34]^	70 days	16	Reduction in severity of articular cartilage erosion; Increased GAG synthesis in OA joints of horses dosed with supplement compared with OA joints in undosed horses	2007
Exercise and exercise plus OA on synovial fluid and serum biomarkers^[33]^	70 days	16	Increased synovial fluid CS846, CPII, GAG, Col CEQ, C1,2C, osteocalcin, Col I in exercised horses. Increased synovial fluid CS846, CPII, GAG, Col CEQ, C1,2C, osteocalcin, Col I and PGE2 in exercise and OA horses *vs.* exercise alone. Increased serum CS846, CPII, GAG, osteocalcin, C1,2C and Col I in exercised horses. Horses with OA and exercise had increased serum CS846, CPII, GAG, osteocalcin, C1,2C and Col I *vs.* exercise alone.	2008
Exercise *vs.* exercise and OA on imaging outcomes^[45]^	70 days	16	Increased radiographic lysis and nuclear scintigraphic uptake in OA/exercise horses *vs.* exercise alone; Increased subchondral bone edema; Bone edema and scintigraphic uptake correlated with Col I and Col II	2008
IA Polysulfated GAG/HA^[46]^	70 days	24	No adverse effects related to treatment; Reduced effusion; Reduced synovial membrane vascularity and subintimal fibrosis in treated joints; Reduced cartilage fibrillation with HA in treated joints	2009
Extracorporeal shock wave therapy^[47]^	70 days	24	No adverse effects associated with treatment; Reduced lameness in treated joints	2009
IA Adipose-derived stromal vascular fraction and BM-MSCs^[48]^	70 days	24	Reduced PGE-2 in treated joints with BM-MSCs; Greater improvement with BM-MSCs	2009
Extracorporeal shock wave therapy (ECSWT) and IM polysulfated glycosaminoglycans^[49]^	70 days	24	Increased serum osteocalcin and C-terminal telopeptide of Col I in joints treated with ECSWT; Synovial fluid CS846 greatest in joints treated with ECSWT	2011
IM administered sodium pentosan polysulfate^[50]^	70 days	18	No adverse effects from treatment; Reduced articular cartilage fibrillation in OA joints in treated horses; Increased synovial fluid chondroitin sulfate 846	2012
Subjective *vs.* objective lameness^[51]^	70 days	16	Reliable correlation between subjective and the inertial sensory system	2015
IV hyaluronan, sodium chondroitin sulfate, and N-acetyl-D-glucosamine^[52]^	70 days	32	Reduced articular cartilage erosion; Increased bone edema in treated joints identified via MRI; Reduced microscopic cartilage abnormalities in OA joints of treated horses	2016
Underwater treadmill (UWT)^[53]^	70 days	16	Reduced synovial membrane inflammation with UWT; Improved symmetric thoracic limb loading, uniform activation patterns in UWT horses; Return to baseline carpal flexion in UWT horses	2017
Oral *Biota orientalis*^[22]^	70 days	16	Reduced PGE-2 in OA joints of treated horses; Reduced radiographic subchondral bone lysis, osteophyte formation, and subchondral sclerosis in the radial carpal bone; Decreased total radiographic score for OA joints of treated horses; No differences in lameness, MRI findings macroscopic or histologic grading in OA joints of treated horses	2022

## Data Availability

Not applicable.

## References

[1] SafiriS, KolahiAA, SmithE, Global, regional and national burden of osteoarthritis 1990–2017: a systematic analysis of the Global Burden of Disease Study 2017. Ann Rheum Dis 2020;79:819–28. DOI3239828510.1136/annrheumdis-2019-216515

[2] ReedSR, JacksonBF, Mc IlwraithCW, Descriptive epidemiology of joint injuries in Thoroughbred racehorses in training. Equine Vet J 2012;44:13–9. DOI2149610310.1111/j.2042-3306.2010.00352.x

[3] WoolfAD. Global burden of osteoarthritis and musculoskeletal diseases. BMC Musculoskelet Disord 2015:16. DOI PMC

[4] KloppenburgM, BerenbaumF. Osteoarthritis year in review 2019: epidemiology and therapy. Osteoarthr Cartil 2020;28:242–8. DOI PubMed10.1016/j.joca.2020.01.00231945457

[5] 2017 Disease and Injury Incidence and Prevalence Collaborators. Global, regional, and national incidence, prevalence, and years lived with disability for 354 diseases and injuries for 195 countries and territories, 1990–2017: a systematic analysis for the Global Burden of Disease Study 2017. Lancet 2018;392:1789–858. DOI PubMed3049610410.1016/S0140-6736(18)32279-7PMC6227754

[6] McilwraithCW. Traumatic arthritis and posttraumatic osteoarthritis in the horse. joint disease in the horse. Elsevier; 2016. p. 33–48. DOI

[7] WangX, HunterDJ, JinX, DingC. The importance of synovial inflammation in osteoarthritis: current evidence from imaging assessments and clinical trials. Osteoarthr Cartil 2018;26:165–74. DOI PubMed10.1016/j.joca.2017.11.01529224742

[8] GoldbergA, MitchellK, SoansJ, KimL, ZaidiR. The use of mesenchymal stem cells for cartilage repair and regeneration: a systematic review. J Orthop Surg Res 2017;12:39. DOI PubMed PMC2827918210.1186/s13018-017-0534-yPMC5345159

[9] KingNM, PerrinJ. Ethical issues in stem cell research and therapy. Stem Cell Res Ther 2014;5:85. DOI PubMed PMC2515742810.1186/scrt474PMC4097842

[10] LooSJQ, WongNK. Advantages and challenges of stem cell therapy for osteoarthritis (Review). Biomed Rep 2021;15:67. DOI PubMed PMC3415545110.3892/br.2021.1443PMC8212446

[11] WangG, XingD, LiuW, Preclinical studies and clinical trials on mesenchymal stem cell therapy for knee osteoarthritis: a systematic review on models and cell doses. Int J Rheum Dis 2022;25:532–62. DOI3524433910.1111/1756-185X.14306

[12] DoyleLM, WangMZ. Overview of extracellular vesicles, their origin, composition, purpose, and methods for exosome isolation and analysis. Cells 2019;8:727. DOI PubMed PMC3131120610.3390/cells8070727PMC6678302

[13] KalraH, DrummenGP, MathivananS. Focus on extracellular vesicles: introducing the next small big thing. Int J Mol Sci 2016;17:170. DOI PubMed PMC2686130110.3390/ijms17020170PMC4783904

[14] LaiRC, ArslanF, LeeMM, Exosome secreted by MSC reduces myocardial ischemia/reperfusion injury. Stem Cell Res 2010;4:214–22. DOI2013881710.1016/j.scr.2009.12.003

[15] BoereJ, MaldaJ, van de LestCHA, van WeerenPR, WaubenMHM. Extracellular vesicles in joint disease and therapy. Front Immunol 2018;9:2575. DOI PubMed PMC3048325510.3389/fimmu.2018.02575PMC6240615

[16] LiK, YanG, HuangH, Anti-inflammatory and immunomodulatory effects of the extracellular vesicles derived from human umbilical cord mesenchymal stem cells on osteoarthritis via M2 macrophages. J Nanobiotechnology 2022;20:38. DOI PubMed PMC3505781110.1186/s12951-021-01236-1PMC8771624

[17] ZhangS, ChuWC, LaiRC, LimSK, HuiJH, TohWS. Exosomes derived from human embryonic mesenchymal stem cells promote osteochondral regeneration. Osteoarthr Cartil 2016;24:2135–40. DOI10.1016/j.joca.2016.06.02227390028

[18] TanSSH, TjioCKE, WongJRY, Mesenchymal stem cell exosomes for cartilage regeneration: a systematic review of preclinical *in vivo* studies. Tissue Eng Part B Rev 2021;27:1–13. DOI3215946410.1089/ten.TEB.2019.0326

[19] McIlwraithCW, FrisbieDD, KawcakCE. The horse as a model of naturally occurring osteoarthritis. Bone Joint Res 2012;1:297–309. DOI PubMed PMC2361066110.1302/2046-3758.111.2000132PMC3626203

[20] KawcakCE, FrisbieDD, TrotterGW, Effects of intravenous administration of sodium hyaluronate on carpal joints in exercising horses after arthroscopic surgery and osteochondral fragmentation. Am J Vet Res 1997;58:1132–40. PubMed9328667

[21] KawcakCE, NorrdinRW, FrisbieDD, TrotterGW, McilwraithCW. Effects of osteochondral fragmentation and intra-articular triamcinolone acetonide treatment on subchondral bone in the equine carpus. Equine Vet J 1998;30:66–71. DOI PubMed945840110.1111/j.2042-3306.1998.tb04090.x

[22] SeabaughKA, BarrettMF, RaoS, McIlwraithCW, FrisbieDD. Examining the effects of the oral supplement biota orientalis in the osteochondral fragment-exercise model of osteoarthritis in the horse. Front Vet Sci 2022;9:858391. DOI PubMed PMC10.3389/fvets.2022.858391PMC919857735720848

[23] FrisbieDD, KawcakCE, BaxterGM, Effects of triamcinolone acetonide on an *in vivo* equine osteochondral fragment exercise mode. Am J Vet Res 1997;29:349–59. PubMed10.1111/j.2042-3306.1997.tb03138.x9306060

[24] FrisbieDD, CrossMW, McilwraithCW. A comparative study of articular cartilage thickness in the stifle of animal species used in human pre-clinical studies compared to articular cartilage thickness in the human knee. Vet Comp Orthop Traumatol 2006;19:142–6. DOI PubMed16971996

[25] BertoniL, Jacquet-GuibonS, BranlyT, An experimentally induced osteoarthritis model in horses performed on both metacarpophalangeal and metatarsophalangeal joints: Technical, clinical, imaging, biochemical, macroscopic and microscopic characterization. PLoS One 2020;15:e0235251. DOI PubMed PMC10.1371/journal.pone.0235251PMC731625632584901

[26] FrisbieD, GhivizzaniC, RobbinsD, EvansH, McIlwraithW. Treatment of experimental equine osteoarthritis by *in vivo* delivery of the equine interleukin-1 receptor antagonist protein. Gene Ther 2002;9:12–20. DOI1185071810.1038/sj.gt.3301608

[27] CookJL, HungCT, KurokiK, Animal models of cartilage repair. Bone Joint Res 2014;3:89–94. DOI PubMed PMC2469575010.1302/2046-3758.34.2000238PMC3974069

[28] CopePJ, OurradiK, LiY, SharifM. Models of osteoarthritis: the good, the bad and the promising. Osteoarthr Cartil 2019;27:230–9. DOI PubMed PMC10.1016/j.joca.2018.09.016PMC635000530391394

[29] WangY, ChenY, WeiY. Osteoarthritis animal models for biomaterial-assisted osteochondral regeneration. Biomater Transl 2022;3:264–79. DOI3684650510.12336/biomatertransl.2022.04.006PMC9947734

[30] ShepherdDE, SeedhomBB. Thickness of human articular cartilage in joints of the lower limb. Ann Rheum Dis 1999;58:27–34. DOI PubMed PMC1034353710.1136/ard.58.1.27PMC1752762

[31] AhernBJ, ParviziJ, BostonR, SchaerTP. Preclinical animal models in single site cartilage defect testing: a systematic review. Osteoarthr Cartil 2009;17:705–13. DOI PubMed10.1016/j.joca.2008.11.00819101179

[32] BroeckxSY, PilleF, BuntinxS, Evaluation of an osteochondral fragment-groove procedure for induction of metacarpophalangeal joint osteoarthritis in horses. Am J Vet Res 2019;80:246–58. DOI PubMed3080120710.2460/ajvr.80.3.246

[33] FrisbieDD, Al-SobayilF, BillinghurstRC, KawcakCE, McIlwraithCW. Changes in synovial fluid and serum biomarkers with exercise and early osteoarthritis in horses. Osteoarthr Cartil 2008;16:1196–204. DOI PubMed10.1016/j.joca.2008.03.00818442931

[34] KawcakCE, FrisbieDD, McIlwraithCW, WerpyNM, ParkRD. Evaluation of avocado and soybean unsaponifiable extracts for treatment of horses with experimentally induced osteoarthritis. Am J Vet Res 2007;68:598–604. DOI PubMed1754269110.2460/ajvr.68.6.598

[35] SimmonsE, BertoneA, WeisbrodeS. Instability-induced osteoarthritis in the metacarpophalangeal joint of horses. Am J Vet Res 1999;60:7–13. PubMed9918142

[36] BolamCJ, HurtigMB, CruzA, McEwenBJ. Characterization of experimentally induced post-traumatic osteoarthritis in the medial femorotibial joint of horses. Am J Vet Res 2006;67:433–47. DOI PubMed1650690510.2460/ajvr.67.3.433

[37] BoyceM, TrumbleT, CarlsonC, GroschenD, MerrittK, BrownM. Non-terminal animal model of post-traumatic osteoarthritis induced by acute joint injury. Osteoarthr Cartil 2013;21:746–55. DOI PubMed PMC10.1016/j.joca.2013.02.653PMC362405923467035

[38] DelcoML, GoodaleM, TaltsJF, Integrin α10β1-Selected Mesenchymal Stem Cells Mitigate the Progression of Osteoarthritis in an Equine Talar Impact Model. Am J Sports Med 2020;48:612–23. DOI PubMed3200407710.1177/0363546519899087

[39] KammJ, NixonA, WitteT. Cytokine and catabolic enzyme expression in synovium, synovial fluid and articular cartilage of naturally osteoarthritic equine carpi. Equine Vet J 2010;42:693–9. DOI PubMed PMC2103979810.1111/j.2042-3306.2010.00140.xPMC4183755

[40] McIlwraithCW, FrisbieDD, KawcakCE, FullerCJ, HurtigM, CruzA. The OARSI histopathology initiative - recommendations for histological assessments of osteoarthritis in the horse. Osteoarthr Cartil 2010;18 Suppl 3:S93–105. DOI PubMed10.1016/j.joca.2010.05.03120864027

[41] FolandJW, McIlwraithCW, TrotterGW, PowersBE, LamarCH. Effect of betamethasone and exercise on equine carpal joints with osteochondral fragments. Vet Surg 1994;23:369–76. DOI PubMed783959510.1111/j.1532-950x.1994.tb00497.x

[42] FrisbieDD, KawcakCE, McIlwraithCW, TrotterGW, PowersBE. Effects of triamcinolone in an equine *in vivo* osteochondral fragment model. Equine Vet J 1996;29:270. PubMed10.1111/j.2042-3306.1997.tb03138.x9306060

[43] FrisbieDD, KawcakCE, BaxterGM, Effects of 6alpha-methylprednisolone acetate on an equine osteochondral fragment exercise model. Am J Vet Res 1998;59:1619–28. PubMed9858417

[44] FrisbieDD, KawcakCE, WerpyNM, ParkRD, McilwraithCW. Clinical, biochemical, and histologic effects of intra-articular administration of autologous conditioned serum in horses with experimentally induced osteoarthritis. Am J Vet Res 2007;68:290–6. DOI PubMed1733101910.2460/ajvr.68.3.290

[45] KawcakCE, FrisbieDD, WerpyNM, ParkRD, McIlwraithCW. Effects of exercise vs experimental osteoarthritis on imaging outcomes. Osteoarthr Cartil 2008;16:1519–25. DOI PubMed10.1016/j.joca.2008.04.01518504148

[46] FrisbieDD, KawcakCE, McilwraithCW, WerpyNM. Evaluation of polysulfated glycosaminoglycan or sodium hyaluronan administered with experimentally induced osteoarthritis. Am J Vet Res 2009;70:203–9. DOI PubMed1923195210.2460/ajvr.70.2.203

[47] FrisbieDD, KawcakCE, McIlwraithCW. Evaluation of the effect of extracorporeal shock wave treatment on experimentally induced osteoarthritis in middle carpal joints of horses. Am J Vet Res 2009;70:449–54. DOI PubMed1933509910.2460/ajvr.70.4.449

[48] FrisbieDD, KisidayJD, KawcakCE, WerpyNM, McIlwraithCW. Evaluation of adipose-derived stromal vascular fraction or bone marrow-derived mesenchymal stem cells for treatment of osteoarthritis. J Orthop Res 2009;27:1675–80. DOI PubMed1954439710.1002/jor.20933

[49] KawcakCE, FrisbieDD, McIlwraithCW. Effects of extracorporeal shock wave therapy and polysulfated glycosaminoglycan treatment on subchondral bone, serum biomarkers, and synovial fluid biomarkers in horses with induced osteoarthritis. Am J Vet Res 2011;72:772–9. DOI PubMed2162752310.2460/ajvr.72.6.772

[50] McIlwraithCW, FrisbieDD, KawcakCE. Evaluation of intramuscularly administered sodium pentosan polysulfate for treatment of experimentally induced osteoarthritis in horses. Am J Vet Res 2012;73:628–33. DOI PubMed2253339310.2460/ajvr.73.5.628

[51] DonnellJR, FrisbieDD, KingMR, GoodrichLR, HausslerKK. Comparison of subjective lameness evaluation, force platforms and an inertial-sensor system to identify mild lameness in an equine osteoarthritis model. Vet J 2015;206:136–42. DOI PubMed2636174910.1016/j.tvjl.2015.08.004

[52] FrisbieDD, McIlwraithCW, KawcakCE, WerpyNM. Efficacy of intravenous administration of hyaluronan, sodium chondroitin sulfate, and N-acetyl-d-glucosamine for prevention or treatment of osteoarthritis in horses. Am J Vet Res 2016;77:1064–70. DOI PubMed2766857710.2460/ajvr.77.10.1064

[53] KingMR, HausslerKK, KawcakCE, Biomechanical and histologic evaluation of the effects of underwater treadmill exercise on horses with experimentally induced osteoarthritis of the middle carpal joint. Am J Vet Res 2017;78:558–69. DOI PubMed2844105410.2460/ajvr.78.5.558

[54] FrisbieD, KingM, NelsonB, GearingD. *In vivo* assessment of anti nerve growth factor administration either systemically or locally using models of joint disease. Osteoarthr Cartil 2017;25:S442. DOI

[55] GoldringMB, OteroM. Inflammation in osteoarthritis. Curr Opin Rheumatol 2011;23:471–8. DOI PubMed PMC2178890210.1097/BOR.0b013e328349c2b1PMC3937875

[56] BrandtKD, DieppeP, RadinEL. Etiopathogenesis of osteoarthritis. Rheum Dis Clin North Am 2008;34:531–59. DOI PubMed1868727110.1016/j.rdc.2008.05.011

[57] LaneNE, BrandtK, HawkerG, OARSI-FDA initiative: defining the disease state of osteoarthritis. Osteoarthr Cartil 2011;19:478–82. DOI PubMed10.1016/j.joca.2010.09.01321396464

[58] LoeserRF, GoldringSR, ScanzelloCR, GoldringMB. Osteoarthritis: a disease of the joint as an organ. Arthritis Rheum 2012;64:1697–707. DOI PubMed PMC2239253310.1002/art.34453PMC3366018

[59] WenhamCY, ConaghanPG. The role of synovitis in osteoarthritis. Ther Adv Musculoskelet Dis 2010;2:349–59. DOI PubMed PMC2287046010.1177/1759720X10378373PMC3383490

[60] Estrada McDermottJ, PezzaniteL, GoodrichL, Role of innate immunity in initiation and progression of osteoarthritis, with emphasis on horses. Animals (Basel) 2021;11:3247. DOI3482797910.3390/ani11113247PMC8614551

[61] ChenY, WeiJ, HuangY, Macrophages in osteoarthritis: pathophysiology and therapeutics. Am J Transl Res 2020;12:261–8. PubMed PMC32051751PMC7013211

[62] SellamJ, BerenbaumF. The role of synovitis in pathophysiology and clinical symptoms of osteoarthritis. Nat Rev Rheumatol 2010;6:625–35. DOI PubMed2092441010.1038/nrrheum.2010.159

[63] SandellLJ, AignerT. Articular cartilage and changes in arthritis. An introduction: cell biology of osteoarthritis. Arthritis Res 2001;3:107–13. DOI PubMed PMC1117811810.1186/ar148PMC128887

[64] van WeerenPR. General anatomy and physiology of joints. Second Edi. Elsevier Inc.; 2015. DOI

[65] DeyleGD, AllisonSC, MatekelRL, Physical therapy treatment effectiveness for osteoarthritis of the knee: a randomized comparison of supervised clinical exercise and manual therapy procedures versus a home exercise program. Physical Therapy 2005;85:1301–17. DOI PubMed16305269

[66] KnutsenG, EngebretsenL, LudvigsenTC, Autologous chondrocyte implantation compared with microfracture in the knee. A randomized trial. J Bone Joint Surg Am 2004;86:455–64. DOI PubMed1499686910.2106/00004623-200403000-00001

[67] ZhuC, WuW, QuX. Mesenchymal stem cells in osteoarthritis therapy: a review. Am J Transl Res 2021;13:448–61. PubMed33594303PMC7868850

[68] ZanottoGM, FrisbieDD. Current joint therapy usage in equine practice: changes in the last 10 years. Equine Vet J 2021;Epub ahead of print. DOI PubMed10.1111/evj.1348934143532

[69] MiglioreA, ProcopioS. Effectiveness and utility of hyaluronic acid in osteoarthritis. Clin Cases Miner Bone Metab 2015;12:31–3. DOI PubMed PMC10.11138/ccmbm/2015.12.1.031PMC446922326136793

[70] GoodrichLR, NixonAJ. Medical treatment of osteoarthritis in the horse - a review. Vet J 2006;171:51–69. DOI PubMed1642758210.1016/j.tvjl.2004.07.008

[71] van GalenG, SaegermanC, Hyldahl LaursenS, Colonic health in hospitalized horses treated with non-steroidal anti-inflammatory drugs - a preliminary study. J Equine Vet Sci 2021;101:103451. DOI PubMed10.1016/j.jevs.2021.10345133993934

[72] LazzaroniM, Bianchi PorroG. Gastrointestinal side-effects of traditional non-steroidal anti-inflammatory drugs and new formulations. Aliment Pharmacol Ther 2004;20 Suppl 2:48–58. DOI PubMed10.1111/j.1365-2036.2004.02037.x15335413

[73] WerneckeC, BraunHJ, DragooJL. The effect of intra-articular corticosteroids on articular cartilage: a systematic review. Orthop J Sports Med 2015;3:2325967115581163. DOI PubMed PMC10.1177/2325967115581163PMC462234426674652

[74] ChenJ, LiJ, LiR, Efficacy and safety of tanezumab on osteoarthritis knee and hip pains: a meta-analysis of randomized controlled trials. Pain Med 2017;18:374–85. DOI PubMed2803497910.1093/pm/pnw262

[75] Auw YangKG, RaijmakersNJ, van ArkelER, Autologous interleukin-1 receptor antagonist improves function and symptoms in osteoarthritis when compared to placebo in a prospective randomized controlled trial. Osteoarthr Cartil 2008;16:498–505. DOI PubMed10.1016/j.joca.2007.07.00817825587

[76] NixonAJ, GrolMW, LangHM, Disease-modifying osteoarthritis treatment with interleukin-1 receptor antagonist gene therapy in small and large animal models. Arthritis Rheumatol 2018;70:1757–68. DOI PubMed3004489410.1002/art.40668

[77] HorwitzEM, Le BlancK, DominiciM, ; International Society for Cellular Therapy. Clarification of the nomenclature for MSC: The International Society for Cellular Therapy position statement. Cytotherapy 2005;7:393–5. DOI PubMed1623662810.1080/14653240500319234

[78] DominiciM, Le BlancK, MuellerI, Minimal criteria for defining multipotent mesenchymal stromal cells. The International Society for Cellular Therapy position statement. Cytotherapy 2006;8:315–7. DOI PubMed1692360610.1080/14653240600855905

[79] Meirelles LdaS, FontesAM, CovasDT, CaplanAI. Mechanisms involved in the therapeutic properties of mesenchymal stem cells. Cytokine Growth Factor Rev 2009;20:419–27. DOI PubMed1992633010.1016/j.cytogfr.2009.10.002

[80] TohWS, FoldagerCB, PeiM, HuiJH. Advances in mesenchymal stem cell-based strategies for cartilage repair and regeneration. Stem Cell Rev Rep 2014;10:686–96. DOI PubMed2486995810.1007/s12015-014-9526-z

[81] Abd-elsayedA. Stem cells for the creatment of knee osteoarthritis: a comprehensive review. Pain Phys 2018;1:229–42. DOI PubMed29871367

[82] MaheshwerB, PolceEM, PaulK, Regenerative potential of mesenchymal stem cells for the treatment of knee osteoarthritis and chondral defects: a systematic review and meta-analysis. Arthroscopy 2021;37:362–78. DOI PubMed3249765810.1016/j.arthro.2020.05.037

[83] FerrisDJ, FrisbieDD, KisidayJD, Clinical outcome after intra-articular administration of bone marrow derived mesenchymal stem cells in 33 horses with stifle injury. Vet Surg 2014;43:255–65. DOI PubMed2443331810.1111/j.1532-950X.2014.12100.x

[84] BroeckxSY, SeysB, SulsM, Equine allogeneic chondrogenic induced mesenchymal stem cells are an effective treatment for degenerative joint disease in horses. Stem Cells Dev 2019;28:410–22. DOI PubMed3062373710.1089/scd.2018.0061PMC6441287

[85] FortierLA, PotterHG, RickeyEJ, Concentrated bone marrow aspirate improves full-thickness cartilage repair compared with microfracture in the equine model. J Bone Joint Surg Am 2010;92:1927–37. DOI PubMed2072013510.2106/JBJS.I.01284

[86] BaraniakPR, McDevittTC. Stem cell paracrine actions and tissue regeneration. Regen Med 2010;5:121–43. DOI PubMed PMC2001769910.2217/rme.09.74PMC2833273

[87] van BuulGM, VillafuertesE, BosPK, Mesenchymal stem cells secrete factors that inhibit inflammatory processes in short-term osteoarthritic synovium and cartilage explant culture. Osteoarthr Cartil 2012;20:1186–96. DOI PubMed10.1016/j.joca.2012.06.00322771777

[88] ChenYC, ChangYW, TanKP, ShenYS, WangYH, ChangCH. Can mesenchymal stem cells and their conditioned medium assist inflammatory chondrocytes recovery? PLoS One 2018;13:e0205563. DOI PubMed10.1371/journal.pone.0205563PMC624891530462647

[89] TimmersL, LimSK, ArslanF, Reduction of myocardial infarct size by human mesenchymal stem cell conditioned medium. Stem Cell Res 2007;1:129–37. DOI PubMed1938339310.1016/j.scr.2008.02.002

[90] KimGB, ShonOJ, SeoMS, ChoiY, ParkWT, LeeGW. Mesenchymal stem cell-derived exosomes and their therapeutic potential for osteoarthritis. Biology (Basel) 2021;10:285. DOI PubMed PMC3391585010.3390/biology10040285PMC8066608

[91] MustonenAM, NieminenP. Extracellular vesicles and their potential significance in the pathogenesis and treatment of osteoarthritis. Pharmaceuticals (Basel) 2021;14:315. DOI PubMed PMC3391590310.3390/ph14040315PMC8065796

[92] BarkholtL, FloryE, JekerleV, Risk of tumorigenicity in mesenchymal stromal cell-based therapies-bridging scientific observations and regulatory viewpoints. Cytotherapy 2013;15:753–9. DOI PubMed2360259510.1016/j.jcyt.2013.03.005

[93] ZhouT, YuanZ, WengJ, Challenges and advances in clinical applications of mesenchymal stromal cells. J Hematol Oncol 2021;14:24. DOI PubMed3357932910.1186/s13045-021-01037-xPMC7880217

[94] PelttariK, WinterA, SteckE, Premature induction of hypertrophy during *in vitro* chondrogenesis of human mesenchymal stem cells correlates with calcification and vascular invasion after ectopic transplantation in SCID mice. Arthritis Rheum 2006;54:3254–66. DOI PubMed1700926010.1002/art.22136

[95] NicolasR, GoodwinG. Isolation and analysis. The chromosomal proteins. Elsevier; 1982. p. 41–68. DOI

[96] GasserO, HessC, MiotS, DeonC, SanchezJC, SchifferliJA. Characterisation and properties of ectosomes released by human polymorphonuclear neutrophils. Exp Cell Res 2003;285:243–57. DOI PubMed1270611910.1016/s0014-4827(03)00055-7

[97] TaylorRC, CullenSP, MartinSJ. Apoptosis: controlled demolition at the cellular level. Nat Rev Mol Cell Biol 2008;9:231–41. DOI PubMed1807377110.1038/nrm2312

[98] ThéryC, WitwerKW, AikawaE, Minimal information for studies of extracellular vesicles 2018 (MISEV2018): a position statement of the International Society for Extracellular Vesicles and update of the MISEV2014 guidelines. J Extracell Vesicles 2018;7:1535750. DOI PubMed PMC10.1080/20013078.2018.1535750PMC632235230637094

[99] ZengZL, XieH. Mesenchymal stem cell-derived extracellular vesicles: a possible therapeutic strategy for orthopaedic diseases: a narrative review. Biomater Transl 2022;3:175–87. DOI3665477510.12336/biomatertransl.2022.03.002PMC9840092

[100] ZhuY, WangY, ZhaoB, Comparison of exosomes secreted by induced pluripotent stem cell-derived mesenchymal stem cells and synovial membrane-derived mesenchymal stem cells for the treatment of osteoarthritis. Stem Cell Res Ther 2017;8:64. DOI PubMed2827918810.1186/s13287-017-0510-9PMC5345222

[101] GorgunC, PalamàMEF, ReverberiD, Role of extracellular vesicles from adipose tissue- and bone marrow-mesenchymal stromal cells in endothelial proliferation and chondrogenesis. Stem Cells Transl Med 2021;10:1680–95. DOI PubMed3448053310.1002/sctm.21-0107PMC8641083

[102] CapomaccioS, CappelliK, BazzucchiC, Equine adipose-derived mesenchymal stromal cells release extracellular vesicles enclosing different subsets of small RNAs. Stem Cells Int 2019;2019:4957806. DOI10.1155/2019/4957806PMC644244331011332

[103] Arévalo-TurrubiarteM, BarattaM, PontiG, ChiaradiaE, MartignaniE. Extracellular vesicles from equine mesenchymal stem cells decrease inflammation markers in chondrocytes *in vitro*. Equine Vet J 2022;54:1133–43. DOI PubMed PMC3474176910.1111/evj.13537PMC9787580

[104] Tofiño-VianM, GuillénMI, Pérez Del CazMD, SilvestreA, AlcarazMJ. Microvesicles from human adipose tissue-derived mesenchymal stem cells as a new protective strategy in osteoarthritic chondrocytes. Cell Physiol Biochem 2018;47:11–25. DOI PubMed2976393210.1159/000489739

[105] CosenzaS, RuizM, ToupetK, JorgensenC, NoëlD. Mesenchymal stem cells derived exosomes and microparticles protect cartilage and bone from degradation in osteoarthritis. Sci Rep 2017;7:16214. DOI PubMed PMC2917666710.1038/s41598-017-15376-8PMC5701135

[106] CosenzaS, ToupetK, MaumusM, Mesenchymal stem cells-derived exosomes are more immunosuppressive than microparticles in inflammatory arthritis. Theranostics 2018;8:1399–410. DOI PubMed2950762910.7150/thno.21072PMC5835945

[107] CapraE, Lange-ConsiglioA. The biological function of extracellular vesicles during fertilization, early embryo-maternal crosstalk and their involvement in reproduction: review and overview. Biomolecules 2020;10:1510. DOI PubMed PMC3315800910.3390/biom10111510PMC7693816

[108] Lange-ConsiglioA, LazzariB, PerriniC, MicroRNAs of equine amniotic mesenchymal cell-derived microvesicles and their involvement in anti-inflammatory processes. Cell Transplant 2018;27:45–54. DOI PubMed2956277610.1177/0963689717724796PMC6434479

[109] Lange-ConsiglioA, TassanS, CorradettiB, Investigating the efficacy of amnion-derived compared with bone marrow-derived mesenchymal stromal cells in equine tendon and ligament injuries. Cytotherapy 2013;15:1011–20. DOI PubMed2360257710.1016/j.jcyt.2013.03.002

[110] LiuH, LiM, ZhangT, Engineered bacterial extracellular vesicles for osteoporosis therapy. Chem Eng J 2022;450:138309. DOI

[111] LiuH, ZhangH, HanY, HuY, GengZ, SuJ. Bacterial extracellular vesicles-based therapeutic strategies for bone and soft tissue tumors therapy. Theranostics 2022;12:6576–94. DOI PubMed3618561310.7150/thno.78034PMC9516228

[112] YinH, LiM, TianG, The role of extracellular vesicles in osteoarthritis treatment via microenvironment regulation. SSRN J 2022:preprint. DOI10.1186/s40824-022-00300-7PMC953282036199125

[113] LiangY, XuX, LiX, Chondrocyte-targeted microRNA delivery by engineered exosomes toward a cell-free osteoarthritis therapy. ACS Appl Mater Interfaces 2020;12:36938–47. DOI PubMed3281439010.1021/acsami.0c10458

[114] GurungS, PerocheauD, TouramanidouL, BaruteauJ. The exosome journey: from biogenesis to uptake and intracellular signalling. Cell Commun Signal 2021;19:47. DOI PubMed PMC3389274510.1186/s12964-021-00730-1PMC8063428

[115] MulcahyLA, PinkRC, CarterDR. Routes and mechanisms of extracellular vesicle uptake. J Extracell Vesicles 2014;3:24641. DOI PubMed PMC10.3402/jev.v3.24641PMC412282125143819

[116] JeyaramA, JaySM. Preservation and storage stability of extracellular vesicles for therapeutic applications. AAPS J 2017;20:1. DOI PubMed PMC2918173010.1208/s12248-017-0160-yPMC6582961

[117] WatsonDC, YungBC, BergamaschiC, Scalable, cGMP-compatible purification of extracellular vesicles carrying bioactive human heterodimeric IL-15/lactadherin complexes. J Extracell Vesicles 2018;7:1442088. DOI10.1080/20013078.2018.1442088PMC584402729535850

[118] PaoliniL, Monguió-tortajadaM, CostaM, Large-scale production of extracellular vesicles: report on the “massivEVs” ISEV workshop. J of Extracellular Bio 2022:1. DOI10.1002/jex2.63PMC1108078438939213

[119] WithrowJ, MurphyC, LiuY, HunterM, FulzeleS, HamrickMW. Extracellular vesicles in the pathogenesis of rheumatoid arthritis and osteoarthritis. Arthritis Res Ther 2016;18:286. DOI PubMed PMC2790603510.1186/s13075-016-1178-8PMC5134070

[120] MurphyC, WithrowJ, HunterM, Emerging role of extracellular vesicles in musculoskeletal diseases. Mol Aspects Med 2018;60:123–8. DOI PubMed2896575010.1016/j.mam.2017.09.006PMC5856577

[121] MaldaJ, BoereJ, van de LestCHA, van WeerenPR, WaubenMHM. Extracellular vesicles - new tool for joint repair and regeneration. Nat Rev Rheumatol 2016;12:243–9. DOI PubMed PMC2672946110.1038/nrrheum.2015.170PMC7116208

[122] LiJJ, Hosseini-BeheshtiE, GrauGE, ZreiqatH, LittleCB. Stem Cell-Derived Extracellular Vesicles for Treating Joint Injury and Osteoarthritis. Nanomaterials (Basel) 2019;9:261. DOI PubMed PMC3076985310.3390/nano9020261PMC6409698

[123] NiZ, KuangL, ChenH, The exosome-like vesicles from osteoarthritic chondrocyte enhanced mature IL-1β production of macrophages and aggravated synovitis in osteoarthritis. Cell Death Dis 2019;10:522. DOI PubMed3128542310.1038/s41419-019-1739-2PMC6614358

[124] NakasaT, MiyakiS, KatoT, TakadaT, NakamuraY, OchiM. Exosome derived from osteoarthritis cartilage induces catabolic factor gene expressions in synovium. ORS, Annual Meeting, San Francisco; 2016.

[125] KatoT, MiyakiS, IshitobiH, Exosomes from IL-1β stimulated synovial fibroblasts induce osteoarthritic changes in articular chondrocytes. Arthritis Res Ther 2014;16:R163. DOI PubMed PMC2509237810.1186/ar4679PMC4261911

[126] HeL, HeT, XingJ, Bone marrow mesenchymal stem cell-derived exosomes protect cartilage damage and relieve knee osteoarthritis pain in a rat model of osteoarthritis. Stem Cell Res Ther 2020;11:276. DOI PubMed3265082810.1186/s13287-020-01781-wPMC7350730

[127] HothamWE, ThompsonC, Szu-TingL, HensonFMD. The anti-inflammatory effects of equine bone marrow stem cell-derived extracellular vesicles on autologous chondrocytes. Vet Rec Open 2021;8:e22. DOI PubMed PMC3479590410.1002/vro2.22PMC8580791

[128] VonkLA, van DooremalenSFJ, LivN, Mesenchymal stromal/stem cell-derived extracellular vesicles promote human cartilage regeneration *in vitro*. Theranostics 2018;8:906–20. DOI PubMed2946399010.7150/thno.20746PMC5817101

[129] HothamWE, ThompsonCH, NewellK, Szu TingL, HensonF. The isolation and characterisation of equine bone marrow stem cell derived extracellular vesicles - evidence of an anti-inflammatory action on chondrocytes. Res Sq; 2020:preprint. DOI

[130] LiuY, ZouR, WangZ, WenC, ZhangF, LinF. Exosomal KLF3-AS1 from hMSCs promoted cartilage repair and chondrocyte proliferation in osteoarthritis. Biochem J 2018;475:3629–38. DOI3034116610.1042/BCJ20180675

[131] ZhangS, ChuahSJ, LaiRC, HuiJHP, LimSK, TohWS. MSC exosomes mediate cartilage repair by enhancing proliferation, attenuating apoptosis and modulating immune reactivity. Biomaterials 2018;156:16–27. DOI PubMed2918293310.1016/j.biomaterials.2017.11.028

[132] ZhangS, WongKL, RenX, Mesenchymal stem cell exosomes promote functional osteochondral repair in a clinically relevant porcine model. Am J Sports Med 2022;50:788–800. DOI3509932710.1177/03635465211068129

[133] YangH, CongM, HuangW, The effect of human bone marrow mesenchymal stem cell-derived exosomes on cartilage repair in rabbits. Stem Cells Int 2022;2022:5760107. DOI PubMed PMC10.1155/2022/5760107PMC947759536117721

[134] WiklanderOP, NordinJZ, O’LoughlinA, Extracellular vesicle *in vivo* biodistribution is determined by cell source, route of administration and targeting. J Extracell Vesicles 2015;4:26316. DOI PubMed PMC2589940710.3402/jev.v4.26316PMC4405624

[135] LeeJY, KimHS. Extracellular vesicles in regenerative medicine: potentials and challenges. Tissue Eng Regen Med 2021;18:479–84. DOI PubMed PMC10.1007/s13770-021-00365-wPMC830006734297340

[136] LenerT, GimonaM, AignerL, Applying extracellular vesicles based therapeutics in clinical trials - an ISEV position paper. J Extracell Vesicles 2015;4:30087. DOI PubMed PMC2672582910.3402/jev.v4.30087PMC4698466

